# Circulating ANGPTL8 levels and risk of kidney function decline: Results from the 4C Study

**DOI:** 10.1186/s12933-021-01317-3

**Published:** 2021-06-24

**Authors:** Huajie Zou, Yongping Xu, Xiaoyu Meng, Danpei Li, Xi Chen, Tingting Du, Yan Yang, Yong Chen, Shiying Shao, Gang Yuan, Xinrong Zhou, Shuhong Hu, Wentao He, Delin Ma, Junhui Xie, Benping Zhang, Jianhua Zhang, Wenjun Li, Zhelong Liu, Xuefeng Yu

**Affiliations:** 1 Division of Endocrinology, Department of Internal Medicine, Tongji Hospital, Tongji Medical College, Huazhong University of Science and Technology, 1095 Jiefang Avenue, Wuhan, 430030 China; 2 Branch of National Clinical Research Center for Metabolic Diseases, Hubei, China; 3 Computer Center, Tongji Hospital, Tongji Medical College, Huazhong University of Science and Technology, Wuhan, China

**Keywords:** ANGPTL8, Kidney function decline, eGFR, 4C Study

## Abstract

**Background:**

ANGPTL8, an important regulator of lipid metabolism, was recently proven to have additional intracellular and receptor-mediated functions. This study aimed to investigate circulating levels of ANGPTL8 and its potential association with the risk of kidney function decline in a cohort study.

**Methods:**

We analysed 2,311 participants aged 40 years old and older from the China Cardiometabolic Disease and Cancer Cohort (4C) Study. Kidney function decline was defined as an estimated glomerular filtration rate (eGFR) less than 60 mL per minute per 1.73 m^2^ of body surface area, a decrease in eGFR of ≥ 30% from baseline, chronic kidney disease (CKD)-related hospitalization or death, or end-stage renal disease. The association between baseline ANGPTL8 levels and kidney function decline was assessed using multivariable-adjusted Cox proportional hazards models, and inverse possibility of treatment weight (IPTW) was utilized to prevent overfitting.

**Results:**

There were 136 (5.9%) cases of kidney function decline over a median of 3.8 years of follow-up. We found that serum ANGPTL8 levels at baseline were elevated in individuals with kidney function decline compared to those without kidney function decline during follow-up (718.42 ± 378.17 vs. 522.04 ± 283.07 pg/mL, p < 0.001). Compared with the first quartile, multivariable-adjusted hazard ratio (95% confidence intervals [CIs]) for kidney function decline was 2.59 (95% CI, 1.41–4.77) for the fourth ANGPTL8 quartile. Furthermore, compared with patients in the first ANGPTL8 quartile, those in the fourth ANGPTL8 quartile were more likely to report a higher stage of CKD (relative risk: 1.33; 95% CI, 1.01–1.74). The conclusions of the regression analyses were not altered in the IPTW models. Multivariable-adjusted restricted cubic spline analyses suggested a linear relationship of ANGPTL8 with kidney function decline (p for nonlinear trend = 0.66, p for linear trend < 0.001).

**Conclusions:**

Participants with higher circulating ANGPTL8 levels were at increased risk for kidney function decline, highlighting the importance of future studies addressing the pathophysiological role of ANGPTL8 in CKD.

**Supplementary Information:**

The online version contains supplementary material available at 10.1186/s12933-021-01317-3.

## Background

Angiopoietin-like protein 8 (ANGPTL8) has been called different names, such as betatrophin, TD26, “refeeding induced in fat and liver” (RIFL), lipasin, and PRO1185 [1]. In the blood, ANGPTL8 forms a complex with ANGPTL3 or ANGPTL4 to inhibit lipoprotein lipase (LPL) and regulate triglyceride (TG) metabolism under fed or fasted conditions, respectively [2]. Recently, evidence has emerged for additional intracellular and receptor-mediated functions of ANGPTL8, with implications in NF-κB-mediated inflammation, autophagy, adipogenesis, intracellular lipolysis and regulation of the circadian clock [3]. Elevated levels of plasma ANGPTL8 are associated with metabolic syndrome [45, 46], type 2 diabetes mellitus (T2DM) [4–6], non-alcoholic fatty liver disease (NAFLD)/non-alcoholic steatohepatitis (NASH) [7, 8], atherosclerosis [9, 10] and hypertension [11]. Furthermore, hypertension and atherosclerosis are important risk factors for future estimated glomerular filtration rate (eGFR) decline and the development of kidney disease [12, 13]***.***

Chronic kidney disease (CKD) is a devastating condition that is reaching epidemic levels owing to the increasing prevalence of diabetes mellitus, hypertension and obesity, as well as the ageing of the population [14]. CKD may progress to irreversible nephron loss, end-stage renal disease and/or premature death [14]. The burden of CKD is substantial. According to WHO global health estimates, CKD accounted for 12.2 deaths per 100,000 people, ranking fourteenth in the list of leading causes of death [15].

Previous cross-sectional studies revealed that ANGPTL8 levels were increased in T2DM patients with albuminuria [16, 17]. Our previous study also found that circulating ANGPTL8 levels were negatively correlated with the eGFR [18], which indicates that ANGPTL8 may play a potential role in the progression of kidney disease. Whether ANGPTL8 is a protective regulator or a risk factor in kidney disease remains to be explored. In this work, we retrospectively investigated the association between circulating ANGPTL8 levels and the risk for developing CKD in a 5-year cohort study.

## Materials and methods

### Study design and population

Participants in the present study were recruited from Hubei Province, China, from 2011 to 2012 as part of the Risk Evaluation of Cancer in Chinese Diabetic Individuals: A Longitudinal (REACTION) Study, which was renamed the China Cardiometabolic Disease and Cancer Cohort (4C) Study [19–22]. The study is a multicentre, population-based, prospective cohort study of Chinese adults aged ≥ 40 years investigating the associations of glucose homeostasis with clinical outcomes, including diabetes, cardiovascular disease (CVD), cancer, and all-cause mortality [23].

Of the 10,999 study participants, 9,221 (83.8%) were followed up during 2011–2016. As shown in Additional file 1: Fig. S1, we excluded participants who were older than 90 years (N = 47), had prevalent CKD (eGFR < 60 mL/min/1.73 m^2^ or CKD-related hospitalization) (N = 245) or missing serum creatinine values at baseline (N = 979), were not fasting at baseline (N = 1,031) or visits (N = 508), were missing body mass index (BMI) values, glucose measurements, glycaemic measures, lipid profiles or information on a history of CVD, diabetes or hypertension (N = 3,142), or had missing outcome data (N = 935). Additionally, the highest and lowest 0.5% of ANGPTL8 values were trimmed (N = 23) [23], leaving 2,311 participants for analysis.

The Committee on Human Research at Ruijin Hospital, Shanghai Jiaotong University School of Medicine, approved the study protocol (Shanghai, China: 14/2004), and all of the participants provided written informed consent. All of the methods were performed in accordance with the relevant guidelines and regulations.

### Laboratory tests of ANGPTL8

Blood samples were collected after overnight fasting. Serum was separated by centrifugation, aliquoted and then stored at  − 80 °C. Fasting serum ANGPTL8 levels were assessed using ELISA kits (Eiaab Science, Wuhan, China; Catalogue No. E11644h) with an intra-assay coefficient of variation (CV) of ≤ 6.5% and an interassay CV of ≤ 9.2% (provided by the manufacturer). The procedures were performed in accordance with the manufacturer’s instructions. All of the samples were analysed in duplicate.

### Outcome assessment

Kidney function decline was defined as meeting at least 1 of the following criteria: (1) eGFR < 60 mL/min/1.73 m^2^ according to the CKD-Epidemiology Collaboration (CKD-EPI) formula [24, 25], (2) a decrease in eGFR of ≥ 30% from baseline, established as a surrogate for the development of kidney failure [26], (3) hospitalization related to CKD stage ≥ 3, (4) death related to CKD stage ≥ 3, or (5) end-stage renal disease (ESRD). The CKD stage was classified based on the eGFR category, which was assigned as follows: (1) G1, eGFR ≥ 90 mL/min/1.73 m^2^; (2) G2, 60 ≤ eGFR < 90 mL/min/1.73 m^2^; (3) G3, 30 ≤ eGFR < 60 mL/min/1.73 m^2^; (4) G4, 15 ≤ eGFR < 30 mL/min/1.73 m^2^; and (5) G5, eGFR < 15 mL/min/1.73 m^2^ [25, 27]. All of these outcomes were confirmed by death certificates and hospital records.

### Statistical analysis

Continuous variables are expressed as the means ± standard deviations (SDs) (for normally distributed data) or medians (interquartile ranges, IQRs) (for asymmetrically distributed data). Normal distribution of the data was tested using the Kolmogorov–Smirnov test. We obtained p values using the Kruskal–Wallis test for continuous variables and the χ^2^ test for categorical variables. We used Cox proportional hazards models to investigate the association between ANGPTL8 levels at baseline and the risk of kidney function decline. The relationship between ANGPTL8 and CKD stage was tested using ordinal logistic regression. We adjusted multivariable models for baseline age, sex, BMI, high-density lipoprotein (HDL), low-density lipoprotein (LDL), TC (total cholesterol), TGs, aspartate aminotransferase (AST), alanine transaminase (ALT), and history of diabetes, hypertension and CVD. The inverse possibility of treatment weight (IPTW) was utilized to prevent overfitting [28]. Stratified analyses were conducted according to age, sex, BMI, diabetes, hypertension, CVD or hyperlipidaemia at baseline. Correlations between variables were assessed using Spearman and partial correlation analyses. Potential nonlinear relationships between the levels of ANGPTL8 and the incidence of clinical outcomes were examined with restricted cubic splines [29]. A knot was located at the 25th, 50th, and 75th percentiles for ANGPTL8. Tests for nonlinearity were conducted using likelihood ratio tests. If a test for nonlinearity was not significant, we conducted a test for linearity. Receiver-operator characteristic (ROC) curves were drawn, and the performance of the model was evaluated by the area under the curve (AUC). A 2-tailed p value < 0.05 was considered significant. SPSS software version 20.0, Stata software version 12.0, and SAS were used for all of the analyses.

## Results

### Baseline characteristics

**Fig. 1:**
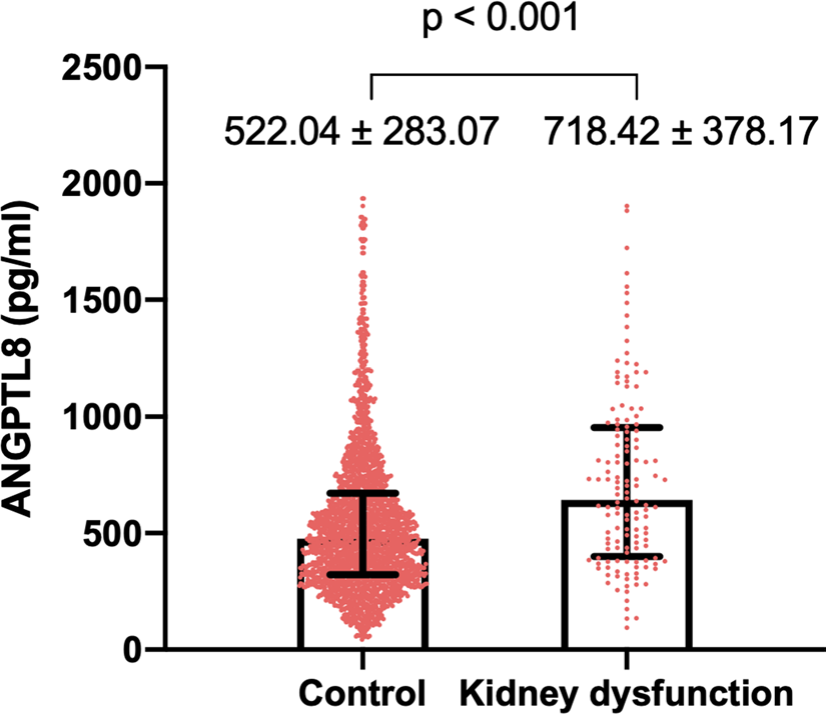
Circulating ANGPTL8 levels in patients with and without kidney function decline during follow-up. The boxplot displays median and IQR

Of the 2,311 participants, 136 (5.9%) patients developed kidney function decline during a median follow-up of 3.8 years (Table 1). We found that serum ANGPTL8 levels at baseline were elevated in individuals with kidney function decline compared with those without kidney function decline during the follow-up (718.42 ± 378.17 vs. 522.04 ± 283.07 pg/mL, p < 0.001, Fig. 1).

**Table 1:** Clinical and biochemical parameters for participants, according to quartile of ANGPTL8 levels

Characteristics	Q1 (187.78–287.78)	Q2 (359.06–436.95)	Q3 (517.94–605.04)	Q4 (751.47–1057.66)	All	P value
N	577	579	578	577	2311	
Age (years)	58 (52–64)	60 (53–67)	63 (56–69)	64 (58–71)	62 (55–68)	< 0.001
Male (%)	135 (23.4)	194 (33.5)	240 (41.5)	261 (45.2)	830 (35.9)	< 0.001
BMI (kg/m^2^)	23.77 (21.67–25.94)	23.34 (21.47–26.14)	23.23 (21.30–25.68)	23.24 (21.17–25.64)	23.43 (21.41–25.81)	0.09
WHR	0.87 (0.83–0.90)	0.87 (0.83–0.90)	0.87 (0.83–0.90)	0.88 (0.83–0.91)	0.87 (0.83–0.90)	0.33
HbA1c (%)	5.70 (5.40–6.00)	5.70 (5.40–6.00)	5.70 (5.50–6.10)	5.70 (5.40–6.20)	5.70 (5.40–6.00)	0.07
FPG (mmol/L)	5.27 (4.92–5.74)	5.22 (4.88–5.71)	5.34 (4.93–6.02)	5.38 (4.94–6.15)	5.28 (4.92–5.91)	< 0.001
2 h PG (mmol/L)	6.18 (5.19 -7.65)	6.32 (5.23–7.68)	6.50 (5.44–8.44)	6.70 (5.55–9.12)	6.43 (5.30–8.16)	< 0.001
HDL (mmol/L)	1.48 (1.26–1.74)	1.44 (1.25–1.70)	1.47 (1.24–1.73)	1.40 (1.17–1.69)	1.45 (1.23–1.71)	0.006
LDL (mmol/L)	2.82 (2.30–3.37)	2.77 (2.27–3.32)	2.80 (2.26–3.33)	2.79 (2.29–3.35)	2.79 (2.28–3.34)	0.88
TG (mmol/L)	1.22 (0.87–1.66)	1.14 (0.81–1.65)	1.08 (0.84–1.63)	1.31 (0.88–1.92)	1.19 (0.85–1.73)	< 0.001
TC (mmol/L)	5.02 (4.35–5.59)	4.94 (4.34–5.58)	4.96 (4.38–5.64)	5.04 (4.32–5.66)	4.99 (4.35–5.62)	0.64
ALT (U/L)	12 (9–17)	12 (9–16)	12 (10–17)	14 (10–19)	13 (10–17)	< 0.001
AST (U/L)	20 (17–24)	21 (18–25)	21 (18–26)	23 (19–28)	21 (18–26)	< 0.001
Creatinine at baseline (μmol/L)	58.50 (54.70–63.45)	59.40 (55.00–64.60)	60.60 (56.10–65.80)	62.30 (56.85–68.30)	60.30 (55.50–65.50)	< 0.001
eGFR at baseline (mL/min/1.73 m^2^)	99.77 (94.23–105.04)	98.52 (93.35–104.31)	96.72 (91.72–102.07)	94.95 (89.45–100.35)	97.36 (92.19–103.05)	< 0.001
Hypertension (%)	300 (52.0)	301 (52.0)	330 (57.1)	341 (59.1)	1272 (55.0)	0.03
Hyperlipidaemia (%)	213 (36.9)	213 (36.8)	211 (36.5)	260 (45.1)	897 (38.8)	0.005
DM (%)	114 (19.8)	102 (17.6)	153 (26.5)	172 (29.8)	541 (23.4)	< 0.001
CVD (%)	27 (4.7)	30 (5.2)	19 (3.3)	33 (5.7)	109 (4.7)	0.24
Kidney function decline (%)	16 (2.8)	29 (5.0)	25 (4.3)	66 (11.4)	136 (5.9)	< 0.001
CKD stage						
G1 (%) (eGFR≥ 90)	382 (66.2)	355 (61.3)	332 (57.4)	253 (43.8)	1322 (57.2)	< 0.001
G2 (%) (60 ≤ eGFR < 90)	183 (31.7)	201 (34.7)	229 (39.6)	274 (47.5)	887 (38.4)	
G3 (%) (30 ≤ eGFR < 60)	11 (1.9)	22 ( 3.8)	16 ( 2.8)	47 ( 8.1)	96 (4.2)	
G4 (%) (15 ≤ eGFR < 30)	1 (0.2)	1 ( 0.2)	1 ( 0.2)	2 (0.3)	5 ( 0.2)	
G5 (%)(eGFR < 15)	0 (0)	0 ( 0)	0 ( 0)	1 ( 0.2)	1 ( 0.04)	

*BMI* body-mass index, *WHR* waist hip rate, *HbA1c* glycated haemoglobin A1c, *FPG* fasting plasma glucose, *2 h PG* 2 h plasma glucose concentration, *HDL* high density lipoprotein, *LDL* low density lipoprotein, *TG* triglycerides, *TC* total cholesterol, *ALT* alanine transaminase, *AST* aspartate aminotransferase, *eGFR* glomerular filtration rate, *DM* diabetes mellitus, *CVD* cardiovascular diseases, *CKD* chronic kidney disease

The baseline characteristics of the included participants according to quartiles of ANGPTL8 are presented in Table 1. Age, sex, fasting plasma glucose (FPG), 2 h plasma glucose concentration (2 h PG), HDL, TG, AST, ALT, creatinine, and eGFR at baseline changed with ANGPTL8 levels (all p values < 0.05). Furthermore, the prevalence of hypertension, hyperlipidaemia and diabetes increased in the highest quartile of ANGPTL8 levels (p < 0.05). There was no significant difference in medication use for patients with diabetes, hypertension or hyperlipidaemia (all p values > 0.05, Additional file 1: Table S1).

### ANGPTL8 correlated with kidney function decline

As shown in Table 1, increasing quartiles of ANGPTL8 levels were associated with elevated incidences of kidney function decline and an increased number of patients with a higher CKD stage (all p values < 0.001). Compared with the first quartile, multivariable-adjusted hazard ratios (HRs) (95% confidence intervals [CIs]) for kidney function decline were 2.59 (95% CI, 1.41–4.77; model 3 in Table 2) and 1.73 (95% CI, 1.27–2.36; IPTW model in Table 2) for the fourth ANGPTL8 quartile. Furthermore, a per-SD increase in ANGPTL8 levels was associated with a 31% higher risk for kidney function decline (HR, 1.31; 95% CI, 1.13–1.51). Additional file 1: Table S2 shows the associations between ANGPTL8 and CKD stage. Adjusted estimates revealed that compared with participants in the first ANGPTL8 quartile, those in the fourth ANGPTL8 quartile were more likely to report a higher stage of CKD (relative risk [RR]: 1.33; 95% CI, 1.01–1.74; model 3 in Additional file 1: Table S2), which was consistent with the results in the IPTW model.

**Table 2:** Relation between ANGPTL8 and kidney function decline, according to quartiles of ANGPTL8

ANGPTL8	Model 1	Model 2	Model 3	IPTW model
Q1 (Reference)	1	1	1	
Q2 (HR, 95% CI)	1.91 (1.04–3.51)	1.86 (0.98–3.54)	1.52 (0.79–2.94)	1.72 (1.10–2.68)
Q3 (HR, 95% CI)	1.74 (0.93–3.25)	1.45 (0.74–2.85)	1.43 (0.73–2.80)	1.36 (0.86–2.14)
Q4 (HR, 95% CI)	4.66 (2.70–8.05)	3.99 (2.21–7.20)	2.59 (1.41–4.77)	1.73 (1.27–2.36)
Per-SD increase	1.64 (1.45–1.85)	1.55 (1.36–1.77)	1.31 (1.13–1.51)	-

Model 1 was unadjusted

Model 2 was adjusted for age, sex and BMI

Model 3 was adjusted for all variables in model 2 plus baseline eGFR, HDL, LDL, TC, TG, ALT, AST, history of diabetes, hypertension and CVD at baseline

*HR* hazards ratio, *CI* confidence intervals, *IPTW* inverse possibility of treatment weight, *SD* standard deviation

After controlling for multiple variables, ANGPTL8 levels were also positively correlated with age (r = 0.24), FPG (r = 0.07), TGs (r = 0.04), AST (r = 0.08) and creatinine (r = 0.13) and inversely correlated with HDL (r =  − 0.05), eGFR at baseline (r =  − 0.13), eGFR at the final visit (r =  − 0.11) and eGFR change ($\Delta$ eGFR%) (r = − 0.07) (all p values < 0.05; Additional file 1: Table S3).

**Fig. 2:**
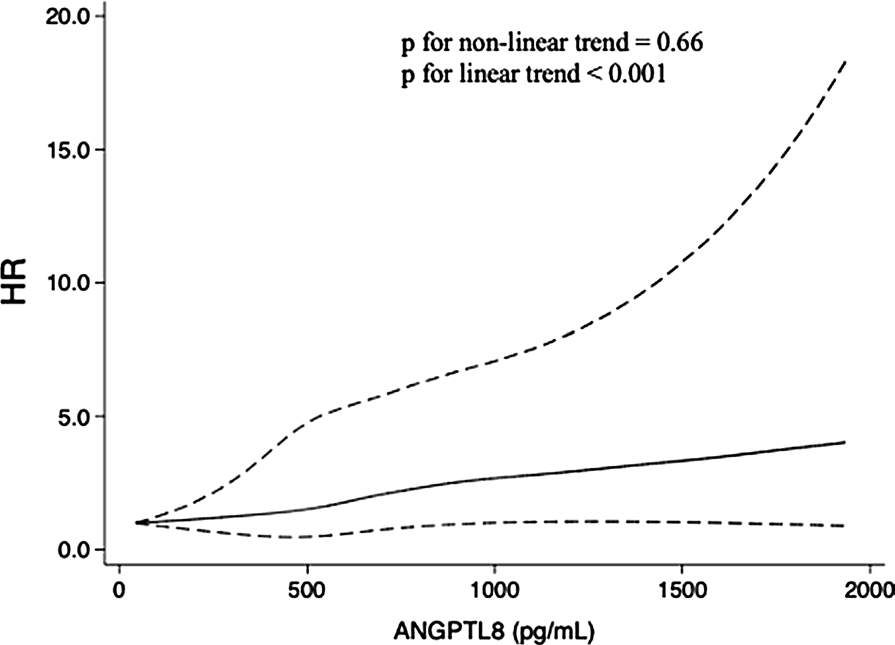
Multivariable-adjusted HRs (95% CI) for kidney function decline. The solid lines indicate multivariate-adjusted HRs, and dotted lines indicate the 95% CIs derived from restricted cubic spline regression. A knot is located at the 25th, 50th, and 75th percentiles for ANGPTL8 levels. The Cox regression was adjusted for sex, age, BMI, FPG, 2 h PG, baseline eGFR, HDL, LDL, TC, TG, ALT, AST, and history of diabetes, hypertension and CVD at baseline

Multivariable-adjusted restricted cubic spline analyses suggested a linear relationship of ANGPTL8 with the HR of kidney function decline (p for nonlinear trend = 0.66, p for linear trend < 0.001; Fig. 2).

The ROC curve shown in Additional file 1: Fig. S2 depicts the performance of ANGPTL8 concentration in detecting kidney function decline. The optimal cut-off point was 608.20 pg/mL. Using this cut-off value, diagnostic efficiency for kidney function decline reached the highest value: the AUC was 0.66 (95% CI, 0.61–0.71, p < 0.001), with sensitivity and specificity of 55.1% and 70.1%, respectively.

### Stratified analyses

**Fig. 3:**
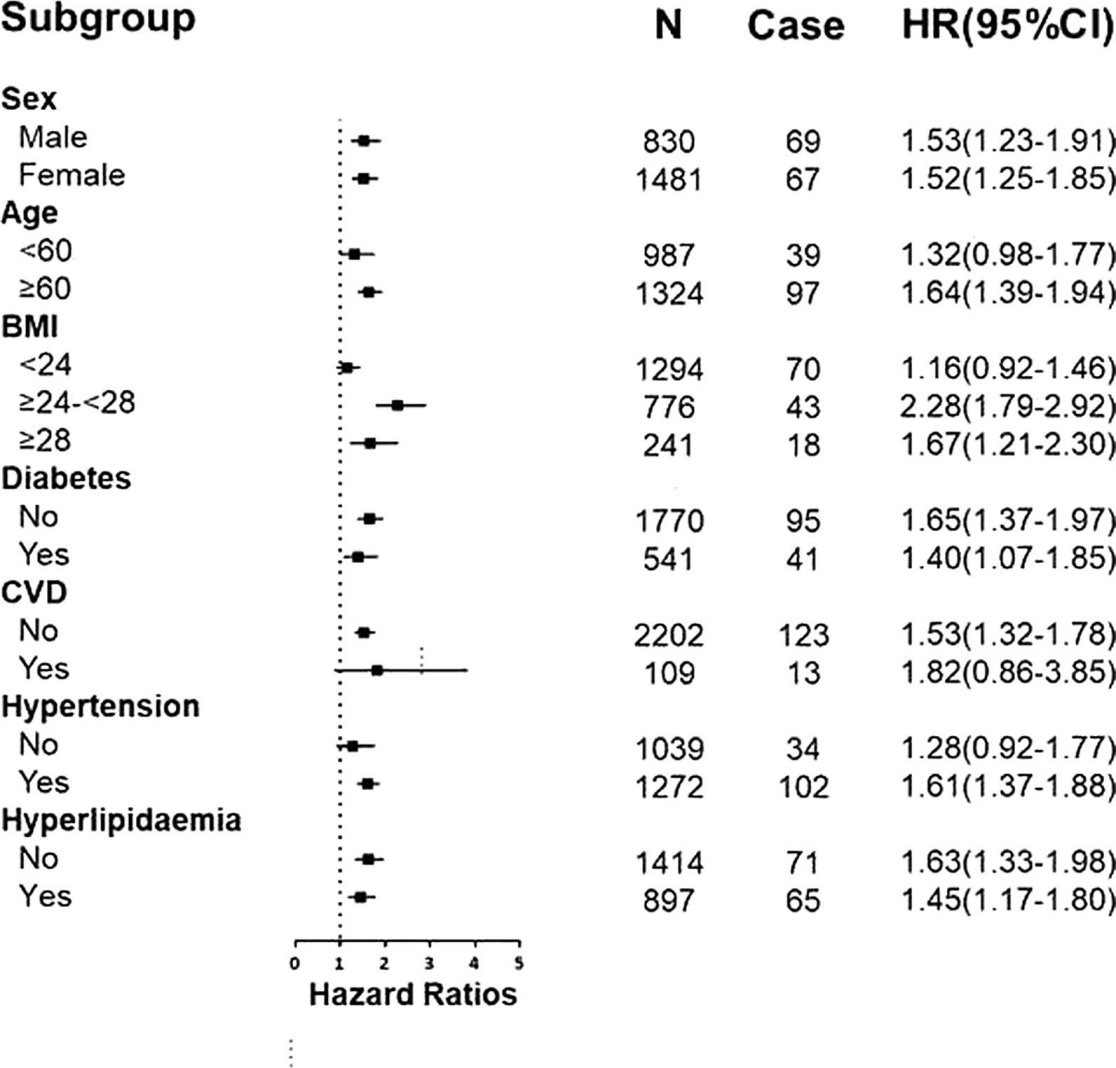
Stratified analyses of the HR of kidney function decline according to a 1-SD increase in ANGPTL8. The final model adjusted for age, sex, BMI, FPG, 2 h PG, HDL, LDL, TC, TG, ALT, AST, and history of diabetes, hypertension and CVD at baseline, except the strata variable

We performed stratified analyses according to age, sex, BMI, diabetes, hypertension, CVD or hyperlipidaemia at baseline. As shown in Fig. 3, the association between ANGPTL8 levels and kidney function decline showed increased significance in individuals with BMI ≥ 24 kg/m^2^, older age (≥ 60 years), hypertension and no CVD (all p value < 0.05). However, the association was diminished in individuals without hypertension or with CVD (all p value > 0.05).

## Discussion

### ANGPTL8 levels correlated with kidney function decline

This retrospective cohort study found that serum baseline ANGPTL8 levels were elevated and independently associated with kidney function decline. Furthermore, multivariable-adjusted restricted cubic spline suggested a dose–response relationship of ANGPTL8 with the risk for kidney function decline. However, estimates for this association in individuals with CVD or without hypertension were not statistically significant.

ANGPTL8, an important regulator of TG metabolism, has recently been proven to have additional intracellular and receptor-mediated functions [3, 30–32]. Previous studies found that ANGPTL8 levels were increased in diabetic nephropathy and associated with eGFR [16, 17, 33, 34]. However, whether ANGPTL8 exerts an effect on the progression of nephropathy or whether it responds to renal dysfunction remains an open question. Nevertheless, our longitudinal study investigated whether ANGPTL8 may be a potential risk factor for CKD.

### Potential mechanisms for the association

The underlying mechanisms for these pathophysiological associations await exposure. Our study found that ANGPTL8 was positively correlated with age, FPG, AST, and TG but inversely correlated with HDL. Previous studies found that ANGPTL8 levels were positively related to age [10, 18, 35]***.*** Stratified analyses also showed that the association between ANGPTL8 levels and kidney function decline was more significant in older individuals (age ≥ 60 years). Age is related to mitochondrial dysfunction, increased oxidative stress, inflammation, and hormonal changes [47], which play an important role in the pathogenesis of CKD progression [14]. Second, the accumulation of lipids in the kidney was shown to promote the progression of renal tubular damage and interstitial fibrosis by leading to inflammation, reactive oxygen species (ROS) production and endogenous electrical stress [36]. Thus, dysregulated lipid metabolism may be the potential mechanism underlying the involvement of ANGPTL8 in the development of CKD. Furthermore, NAFLD was reported to be a driver of CKD [37, 38]. ANGPTL8, a novel hepatokine, was increased in NAFLD [7] and positively correlated with AST. Therefore, ANPTL8 may be a potential link between NAFLD and CKD. Finally, ANGPTL8 has been shown to negatively regulate NF-κB, a key transcription factor implicated in the inflammatory signalling cascade [32]. However, circulating levels of ANGPTL8 have been reported to be elevated in diseases associated with inflammation, such as T2DM [4–6], atherosclerosis [10], and NASH [8]. In addition, plasma levels of ANGPTL8 have been found to be elevated in patients with severe infections, and a strong correlation between circulating ANGPTL8 and the lipopolysaccharide-induced acute inflammatory response in animal models has been observed [32]. Thus, Abu-Farha et al. suggested that even though intracellular ANGPTL8 may prevent the activation of NF-κB, the extracellular actions of circulating ANGPTL8 may cause inflammation [3], which plays a key role in CKD. However, estimates of the association between ANGPTL8 and kidney function decline in individuals with CVD or without hypertension were not statistically significant. The null association in patients with CVD may be due to the relatively small number of events, small sample size (13/109) and correspondingly low power, although there was a trend showing that patients with CVD had a decline in kidney function in numbers similar to patients without CVD. Hypertension status is an important risk factor for future eGFR decline and the development of kidney disease [13], which may be due to higher intraglomerular pressure and progressive arteriosclerosis [12, 39, 40]. Elevated ANGPTL8 levels have been reported to be associated with atherosclerosis [9, 10] and hypertension [11]. Therefore, ANGTPTL8 may contribute to the acceleration of the atherogenic process and renal artery stenosis in patients with hypertension, leading to eGFR decline and the development of CKD.

On the other hand, ANGPTL8 shares considerable structural similarity with ANGPTL3 and ANGPTL4 [41]. As the structure generally determines the function, ANGPTL8 may play a similar role to ANGPTL4. Previous studies suggested that ANGPTL4 is a critical link between proteinuria and hypertriglyceridaemia [42]. Furthermore, podocytes secrete ANGPTL4 and mediate proteinuria in glucocorticoid-sensitive nephrotic syndrome [43]. Therefore, we speculated that podocytes or other kidney cells may also secrete ANGPTL8 in a pathological state and be involved in the pathogenesis of CKD. Above all, further experiments are needed to elucidate the pathophysiological significance of this finding.

## Limitations

There are some limitations of this study. First, all of the participants in our study were Chinese adults aged ≥ 40 years, limiting the generalizability of the findings. Interestingly, the previous 4 cross-sectional studies enrolled populations with a mean age > 50 years and concluded that elevated ANGPTL8 levels were associated with kidney function decline [16, 17, 33, 34], especially in diabetic patients [16, 17, 33]. Among the above studies, 2 were conducted in Asian populations [16, 17], 1 was conducted in an European population [34] and 1 was conducted in an African population [33]. ANGPTL8 levels were reported to vary over a 150-fold range and differed between racial/ethnic groups (Blacks > Hispanics > Whites) [44]. Therefore, the association should be confirmed in other ethnic and age groups. Second, the retrospective design might also have carried the risk of selection bias; therefore, prospective studies are needed to confirm the results. Regardless, this study is the first longitudinal study showing that ANGPTL8 could be a potential risk factor for CKD. Third, we did not have albuminuria data. Therefore, we cannot properly address the association between ANGPTL8 and urinary albumin excretion or the albumin/creatinine ratio as additional markers for kidney function. Finally, the relatively short follow-up duration reduced the number of clinical events and the study’s statistical power, especially in stratified analyses. Future studies should be conducted with larger sample sizes to verify the findings of our study.

## Conclusion

In conclusion, participants with higher circulating ANPTL8 levels had an increased risk for kidney function decline, highlighting the importance of future studies addressing the pathophysiological role of ANGPTL8 in CKD.

## Supplementary Information

**Additional file 1: Table S1.** Medication for patients with chronic diseases, according to quartile of ANGPTL8 levels. **Table S2.** Ordinal logistic regression analysis of the association between ANGPTL8 and renal stage. **Table S3.** Correlations between ANGPTL8 levels and clinical variables. **Figure S1.** Flow diagram for the study population selection. **Figure S2**. ROC curves for the performance of ANGPTL8 concentration in detecting kidney function decline.

## Data Availability

The datasets used and/or analysed during the current study are available from the corresponding author on reasonable request.

## Abbreviations

ALT: Alanine transaminase
ANGPTL8: Angiopoietin-like protein 8
AST: Aspartate aminotransferase
BMI: Body mass index
CI: Confidence intervals
CKD: Chronic kidney disease
CV: Coefficient of variation
CVD: Cardiovascular disease
DM: Diabetes mellitus
eGFR: Estimated glomerular filtration rate
ESRD: End-stage renal disease
FPG: Fasting plasma glucose
2 h PG: 2 H plasma glucose concentration
HbA1c: Glycated haemoglobin A1c
HR: Hazard ratios
HOMA-${\upbeta }$: Homeostasis model assessment of β cell function
HOMA-IR: Homeostasis model assessment of insulin resistance
HDL: High-density lipoprotein
IPTW: Inverse possibility of treatment weight
IQR: Interquartile range
NAFLD: Non- alcoholic fatty liver disease
NASH: Non-alcoholic steatohepatitis
LDL: Low-density lipoprotein
RR: Relative risk
SD: Standard deviation
TC: Total cholesterol
TG: Triglycerides
T2DM: Type 2 diabetes mellitus
WHR: Waist-hip ratio
