# Supplementary material for: Circulating ANGPTL8 levels and risk of kidney function decline: Results from the 4C Study
Source: Cardiovasc Diabetol. 2021 Jun 24;20:127. doi: 10.1186/s12933-021-01317-3 (PMC8223309; doi:10.1186/s12933-021-01317-3)
Supplement: Supplementary file 1 — Additional file 1: Table S1. Medication for patients with chronic diseases, according to quartile of ANGPTL8 levels. Table S2. Ordinal logistic regression analysis of the association between ANGPTL8 and renal stage. Table S3. Correlations between ANGPTL8 levels and clinical variables. Figure S1. Flow diagram for the study population selection. Figure S2. ROC curves for the performance of ANGPTL8 concentration in detecting kidney function decline. [file 12933_2021_1317_MOESM1_ESM.docx]

**Supplementary Online Content**

Huajie Zou, Yongping Xu, Xiaoyu Meng, et al. Circulating ANGPTL8 levels and risk of kidney function decline: results from the 4C Study.

Table S1. Medication for patients with chronic diseases, according to quartile of ANGPTL8 levels.

Table S2. Ordinal logistic regression analysis of the association between ANGPTL8 and renal stage.

Table S3. Correlations between ANGPTL8 levels and clinical variables.

Figure S1. Flow diagram for the study population selection.

Figure S2. ROC curves for the performance of ANGPTL8 concentration in detecting kidney function decline.

***Correspondence**:

Xuefeng Yu, M.D., Ph.D., Professor of Medicine, Division of Endocrinology, Department of Internal Medicine, Tongji Hospital, Tongji Medical College, Huazhong University of Science and Technology, 1095 Jiefang Avenue, Wuhan 430030, China

Email: xfyu188@163.com

Tel./Fax: +86 027 83662883.

Zhelong Liu, M.D., Ph.D., Division of Endocrinology, Department of Internal Medicine, Tongji Hospital, Tongji Medical College, Huazhong University of Science and Technology, 1095 Jiefang Avenue, Wuhan 430030, China.

Email:

Tel./Fax: +86 027 83662883.

**Table S1. Medication for the patients with chronic diseases, according to quartile of ANGPTL8 levels.**

| Medication for chronic diseases | Q1 (187.78-287.78) | Q2 (359.06-436.95) | Q3 (517.94-605.04) | Q4 (751.47-1057.66) | All | P value |
| --- | --- | --- | --- | --- | --- | --- |
| **Patients with diabetes** | 114 | 102 | 153 | 172 | 541 |  |
| Treatment for diabetes (%) | 16 (14.0) | 18 (17.6) | 31 (20.3) | 35 (20.3) | 100 (18.5) | 0.52 |
| Insulin (%) | 1 (0.9) | 3 (2.9) | 5 (3.3) | 5 (2.9) | 14 (2.6) | 0.63 |
| Biguanides (%) | 5 (4.4) | 5 (4.9) | 12 (7.8) | 13 (7.6) | 35 (6.5) | 0.56 |
| Sulfonylureas (%) | 6 (5.3) | 8 (7.8) | 12 (7.8) | 15 (8.7) | 41 (7.6) | 0.75 |
| Meglitinides (%) | 0 (0) | 0 (0) | 0 (0) | 1 (0.6) | 1 (0.2) | 0.54 |
| Alpha-glucosidase inhibitors (%) | 2 (1.8) | 0 (0) | 1 (0.7) | 4 (2.3) | 7 (1.3) | 0.33 |
| Thiazolidinediones (%) | 1 (0.9) | 0 (0) | 1 (0.7) | 0 (0) | 2 (0.4) | 0.54 |
| Others |  |  |  |  |  |  |
| **Patients with hypertension** | 300 | 301 | 330 | 341 | 1272 |  |
| Treatment for hypertension (%) | 88 (29.3) | 82 (27.2) | 101 (30.6) | 108 (31.7) | 379 (29.8) | 0.65 |
| Calcium antagonists (%) | 46 (15.3) | 42 (14.0) | 48 (14.5) | 46 (13.5) | 182 (14.3) | 0.92 |
| Beta-blockers (%) | 1 (0.3) | 2 (0.7) | 0 (0) | 2 (0.6) | 5 (0.4) | 0.53 |
| Diuretics (%) | 10 (3.3) | 7 (2.3) | 8 (2.4) | 13 (3.8) | 38 (3.0) | 0.63 |
| ACEI/ARB (%) | 12 (4.0) | 9 (3.0) | 10 (3.0) | 12 (3.5) | 43 (3.4) | 0.89 |
| Others (%) | 38 (12.7) | 34 (11.3) | 50 (15.2) | 53 (15.5) | 175 (13.8) | 0.35 |
| **Patients with hyperlipidaemia** | 213 | 213 | 211 | 260 | 897 |  |
| Treatment for hyperlipidaemia (%) | 2 (0.9) | 1 (0.5) | 1 (0.5) | 2 (0.8) | 6 (0.7) | 0.91 |

Aberration: ACEI: angiotensin-converting enzyme inhibitors; ARB, angiotensin receptor blocker.

**Table S2. Ordinal logistic regression analysis of the association between ANGPTL8 and CKD stage.**

| ANGPTL8 | Model 1 | Model 2 | Model 3 | IPTW model |
| --- | --- | --- | --- | --- |
| Q1 (Reference) | 1 | 1 | 1 | 1 |
| Q2 (RR, 95% CI) | 1.26 (0.99-1.60) | 1.06 (0.81-1.40) | 1.01 (0.77-1.33) | 0.85 (0.70-1.04) |
| Q3 (RR, 95% CI) | 1.44 (1.14-1.82) | 0.92 (0.70-1.22) | 0.81 (0.61-1.07) | 0.78 (0.64-0.94) |
| Q4 (RR, 95% CI) | 2.63 (2.08-3.33) | 1.64 (1.25-2.16) | 1.33 (1.01-1.74) | 1.27 (1.06-1.52) |

Model 1 was unadjusted.

Model 2 was adjusted for age, sex and BMI.

Model 3 was adjusted for all variables in model 2 plus HDL, LDL, cholesterol, TG, ALT, AST, history of diabetes, hypertension and CVD at baseline.

Abbreviation: RR, relative risk; CI, confidence intervals; IPTW, inverse possibility of treatment weight.

**Table S3. Correlations between ANGPTL8 levels and clinical variables.**

|  | Model 1 | | Model 2 | | Model 3 | |
| --- | --- | --- | --- | --- | --- | --- |
|  | r | p value | Partial r | p value | Partial r | p value |
| Age | 0.267 | 0.000 | 0.235 | 0.000 | 0.242 | 0.000 |
| BMI | -0.052 | 0.012 | 0.033 | 0.115 | 0.001 | 0.965 |
| WHR | 0.042 | 0.046 | 0.015 | 0.485 | 0.010 | 0.634 |
| HbA1c | 0.049 | 0.000 | 0.098 | 0.000 | -0.031 | 0.147 |
| FPG | 0.079 | 0.000 | 0.147 | 0.000 | 0.070 | 0.001 |
| 2h PG | 0.122 | 0.000 | 0.130 | 0.000 | 0.005 | 0.832 |
| HDL | -0.064 | 0.002 | -0.108 | 0.000 | -0.054 | 0.010 |
| LDL | -0.013 | 0.520 | -0.014 | 0.523 | -0.020 | 0.345 |
| TG | 0.044 | 0.034 | 0.145 | 0.000 | 0.043 | 0.044 |
| TC | 0.010 | 0.635 | 0.018 | 0.404 | 0.013 | 0.534 |
| ALT | 0.076 | 0.000 | 0.083 | 0.000 | -0.036 | 0.093 |
| AST | 0.164 | 0.000 | 0.096 | 0.000 | 0.083 | 0.000 |
| Creatinine at baseline | 0.182 | 0.000 | 0.162 | 0.000 | 0.125 | 0.000 |
| eGFR at baseline | -0.227 | 0.000 | -0.166 | 0.000 | -0.126 | 0.000 |
| eGFR at visit | -0.158 | 0.000 | -0.140 | 0.000 | -0.107 | 0.000 |
| $\Delta$eGFR% | -0.058 | 0.005 | -0.082 | 0.000 | -0.071 | 0.001 |

* when a variable was calculated in the partial correlation, it would not be included in adjustment model.

Model 1 was unadjusted.

Model 2 was adjusted for age, sex and BMI.

Model 3 was adjusted for all variables in model 2 plus FPG, 2h PG, HDL, LDL, TC, TG, ALT, AST, eGFR, history of diabetes, hypertension and CVD at baseline.

Abbreviation: BMI, body-mass index; WHR, waist hip rate; HbA1c, glycated haemoglobin A1c; FPG, fasting plasma glucose; 2h PG, 2 h plasma glucose concentration; HOMA-IR, homeostasis model assessment of insulin resistance; HOMA-$\beta$, homeostasis model assessment of β cell function; HDL, high density lipoprotein; LDL, low density lipoprotein; TG, triglycerides; TC, total cholesterol; AST, alanine transaminase; AST aspartate aminotransferase; eGFR, glomerular filtration rate.

**Figure S1. Flow diagram for the study population selection.**


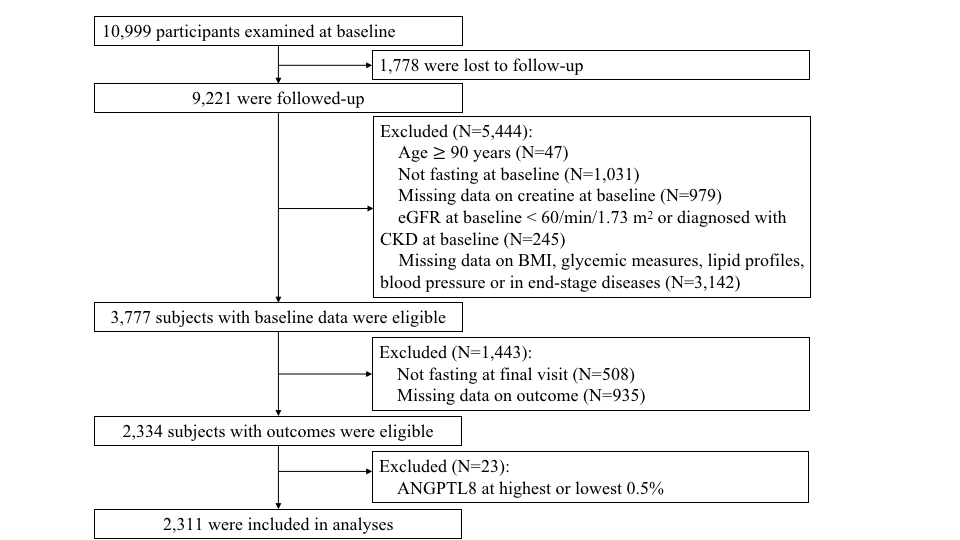


Figure S2. ROC curves for the performance of ANGPTL8 concentration in detecting kidney function decline.


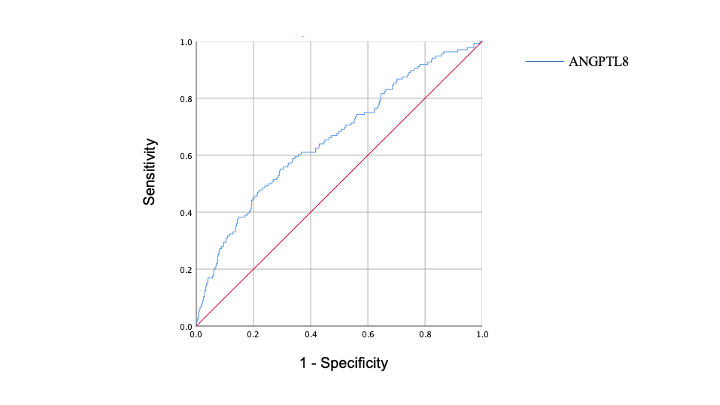


The optimal cut-off point was 608.20 pg/mL. The AUC was 0.66 (95% CI, 0.61-0.71, p < 0.001). The sensitivity and specificity are 55.1% and 70.7%, respectively.
